# New York Veterinary Medical Association

**Published:** 1895-06

**Authors:** 


The New York State Veterinary Medical Association will hold a special meeting this month to take suitable action relative to the Board of Regents' Bill recently passed by the State Legislature, which will regulate the practice of veterinary surgery in New York State.

